# Major latex protein-like encoding genes contribute to *Rhizoctonia solani* defense responses in sugar beet

**DOI:** 10.1007/s00438-020-01735-0

**Published:** 2020-10-28

**Authors:** Louise Holmquist, Fredrik Dölfors, Johan Fogelqvist, Jonathan Cohn, Thomas Kraft, Christina Dixelius

**Affiliations:** 1 MariboHilleshög Research AB, Säbyholmsvägen 24, 26191 Landskrona, Sweden; 2 Department of Plant Biology, Uppsala BioCenter, Linnean Center for Plant Biology, Swedish University of Agricultural Sciences, P.O. Box 7080, 75007 Uppsala, Sweden; 3 Syngenta, Crop Protection, 9 Davis Drive, Research Triangle Park, NC 27709 USA

**Keywords:** Arabidopsis, Defense genes, Major latex protein-like, *Rhizoctonia solani*, RNA-seq, Sugar beet

## Abstract

Sugar beets are attacked by several pathogens that cause root damages. Rhizoctonia (Greek for “root killer”) is one of them. Rhizoctonia root rot has become an increasing problem for sugar beet production and to decrease yield losses agronomical measures are adopted. Here, two partially resistant and two susceptible sugar beet genotypes were used for transcriptome analysis to discover new defense genes to this fungal disease, information to be implemented in molecular resistance breeding. Among 217 transcripts with increased expression at 2 days post-infection (dpi), three resistance-like genes were found. These genes were not significantly elevated at 5 dpi, a time point when increased expression of three Bet v I/Major latex protein (MLP) homologous genes *BvMLP1, BvMLP2* and *BvML3* was observed in the partially resistant genotypes. Quantitative RT-PCR analysis on diseased sugar beet seedlings validated the activity of *BvMLP1* and *BvMLP3* observed in the transcriptome during challenge by *R. solani*. The three *BvMLP* genes were cloned and overexpressed in *Arabidopsis thaliana* to further dissect their individual contribution*.* Transgenic plants were also compared to T-DNA mutants of orthologous *MLP* genes. Plants overexpressing *BvMLP1* and *BvMLP3* showed significantly less infection whereas additive effects were seen on *Atmlp1/Atmlp3* double mutants. The data suggest that *BvMLP1* and *BvMLP3* may contribute to the reduction of the Rhizoctonia root rot disease in sugar beet. Impact on the defense reaction from other differential expressed genes observed in the study is discussed.

**Electronic supplementary material:**

The online version of this article (10.1007/s00438-020-01735-0) contains supplementary material, which is available to authorized users.

## Introduction

*Beta vulgaris* ssp. *vulgaris*, commonly known as sugar beet, is a dicot crop grown in the temperate zone with Europe and the USA as the major production regions (Draycott 2006). The crop is cultivated for its carbohydrate-enriched taproot. In addition to sugar, sugar beet is also a source for an array of carbohydrate-based products including biofuel (Duraisam et al. 2017) and pharmaceuticals such as blood substitute (Leiva-Eriksson et al. 2014). Sugar beet is a biennial crop where carbon is translocated from the leaves to the root during the vegetative stage and vice versa during the generative phase (Fondy et al. 1989). Root crops such as sugar beet that have a relatively long growing season are particularly vulnerable to pathogens including soil microbes attracted to the carbohydrate enriched root system. The soil-borne basidiomycete *Rhizoctonia solani* (teleomorph: *Thanatephorus cucumeris*) has become a pathogen of increasing importance on sugar beet. In the current study, our attempt was to identify defense genes against *R. solani* by comparing transcriptome profiles of sugar beet breeding lines known to express a differential response to this fungal pathogen.

Most *R. solani* infections are initiated by germinating sclerotia or mycelia from debris which can survive in the soil for many years (Cubeta and Vilgalys 1997). Overwintered propagules of *R. solani* germinate and start to infect sugar beet seedlings when soil temperature exceeds 12 °C (Mukhopadhyay 1987). Under optimal temperature and high humidity conditions hyphae colonize the host plant leading to seedling damping-off, crown and root rot (Sneh et al. 1996). *R. solani* AG2-2IIIB is the anastomosis group causing most problems in sugar beet production and soil inoculum is expected to increase in regions where sugar beet and maize are overlapping in the crop rotation schemes, since maize can act as a host and thus propagate the pathogen (Buddemeyer et al. 2004; Schulze et al. 2016). Further, this fungal pathogen does not produce any asexual spores and only occasionally sexual spores are formed (Cubeta and Vilgalys 1997). This lack of spore formation hampers resistance screening work because amounts of inoculum cannot be precisely controlled in field trials or when running indoor experiments. Together all these factors add to the complexity of *R. solani* disease control and work on crop improvement. The strict European regulation on use of agrochemicals prohibits treatment of the soil or the canopy to decrease *R. solani*-incited damages. The only way known to handle the disease is by implementing various cultivation practices and most importantly is the availability of resistant varieties (Buhre et al. 2009). Much work on crop improvements is presently devoted on genomic selection or marker-associated breeding where in this case the sugar beet genome is an important resource (Dohm et al. 2014; Funk et al. 2018).

Based on our transcriptome analysis, we found three major latex protein (MLP) encoding genes *BvMLP1* and *BvMLP2* and *BvMLP3* that showed elevated transcriptional activity in partly resistant genotypes of sugar beet 5 days post-inoculation with *R. solani.* Quantitative RT-PCR confirmed the *BvMLP1* and *BvMLP3* expression in infected sugar beets. Enhanced resistance against *R. solani* infection was also demonstrated when *BvMLP1* and *BvMLP3* were cloned and overexpressed in *A. thaliana*. To dissect individual contributions of the three *MLP* genes, we screened homologous T-DNA mutants in *A. thaliana*. The result showed that both *MLP1* and *MLP3* are of importance in the response to *R. solani*.

## Materials and methods

### Sugar beet material and *R. solani* inoculation for RNAseq

Two partially resistant (G1, line no. 11014044 09; G2, line no. 06012609 70) and two susceptible (G3, line no. 11014038 09; G4, line no. 11014072 09) sugar beet breeding-lines were used. After 13 weeks, the plants were inoculated with *R. solani* AG2-2IIIB BBA 69670 isolate by putting four infected barley kernels approximately 1 cm from the root and 1.5 cm down in the soil on four sides of the root using a tweezers. Inoculated plants were moved from 18/12 °C (day/night) regime to 24/18 °C for the infection phase. At least three roots per genotype were collected before onset of infection (day 0), and 2 and 5 days post-infection (dpi). This experimental design was chosen because it was shown in a pilot study that the fungus reaches the root 2 dpi and we estimated the infection to be in its initial phase at 5 dpi. Further, this experimental design enriches for fungal-induced genes after the inoculation procedure, and reduces the number of development-associated genes in the datasets. Roots were washed and four samples from each root were taken with a core drill. The samples were directly frozen in liquid nitrogen and stored at − 80 °C. In parallel to the infected materials, four roots from each line were harvested before inoculation as control materials.

### RNA isolation

RNA samples were extracted from all four sugar beet genotypes. Three replicates for each time point, treatment and genotype were prepared. Frozen tissue was ground in a mortar to fine powder. Total RNA was isolated according to the procedure outlined by Puthoff and Smigocki (2007) and stored at − 80 °C until further use.

### RNA sequencing and genome mapping

Thirty-six pair end libraries with 100 bp read length were prepared and sequenced using Illumina HiSeq 2000 technology, which generated > 20 million reads per sample. The reads were aligned using GSNAP (genome short-read nucleotide alignment) to the sugar beet genome RefBeet-1.0/Dec 2011 scaffold assembly of KWS2320. Gene IDs were translated to the RefBeet-1.1 version available at https://bvseq.molgen.mpg.de/index.shtml. Count data were generated from BAM files using standard procedures established at National Center for Genome Resources (NCGR), New Mexico, USA. Reads were apportioned (Young et al. 2011) at the gene level to avoid potential data loss associated with using only uniquely aligning reads.

### Data quality control and normalization

Data were evaluated for numbers of read counts for each gene in the samples. A threshold of at least five read counts in each set of three replicates was set. This approach generated a total of 16,768 genes for further analysis. The remaining data sets were manually checked for correct biological affiliation. The quality of samples and major sources of variance were analyzed using multivariate analysis. Data were centered and scaled to unit variance and analyzed by principal component analysis (PCA) in Simca version 13.0.0 (https://umetrics.com/products/simca). Nucleotide percentage by position, average quality (Phred) score by position and bias due to gene length was determined and count data were normalized using the R (version 3.2.3) library EDAseq (Risso et al. 2011).

### Differentially expressed genes (DEG) and gene ontology (GO) enrichment analysis

Differential gene expression analysis was performed using generalized linear model methods (GLMs) implemented in the edgeR package (McCarthy et al. 2012). Absolute log2 fold change > 1 and a false discovery rate (FDR) < 0.05 settings were used to define the DEGs. A heat map was constructed using the pheatmap tool implemented in the R package (Kolde 2015). The R package topGO (Alexa and Rahnenfuhrer 2010) was used for gene ontology (GO) enrichment analysis and functional characterization of the biological processes. Fisher weight or fisherweight01 was used for statistical significance measure with a significance level of < 0.05.

### Co-expression networks and visualization with Cytoscape

Expression data for the 36 samples (4 genotypes, 3 time-points, 3 biological replicates) and 16,768 genes were used to construct weighted gene correlation networks using the WGCNA tool in R-package (Langfelder and Horvath 2008, 2012). Expression count data were converted to log2 + 1, power = 12, TOMtype = unsigned, minModuleSize = 20, reassignThreshold = 1, mergeCutHeight = 0.15, and verbose = 3. Nodes represent genes and edges are correlation coefficient values among gene pair. The network was visualized using Cytoscape version 3.3.0.

### Identification and analysis of carbohydrate-related proteins

Carbohydrate active enzymes (CAZymes) in the sugar beet proteome were analyzed using the dbCAN “Data-Base for automated Carbohydrate-active enzyme Annotation” annotation pipeline (Yin et al. 2012).

### Transgenic *A. thaliana* (*At*) materials

Total RNA was isolated from *B. vulgaris* G1 genotype (Qiagen RNeasy plant mini kit), cDNA was synthesized (qScript™ cDNA synthesis kit, Quanta Biosciences) and used as template for *MLP* gene amplifications. Sugar beet is denoted Bv. Three MLP-like protein encoding genes, *BvMLP1* (Bv7_162510_pymu), *BvMLP2*, (Bv7_162520_etow) and *BvMLP*3 (Bv_27270_xeas) were amplified (Phusion High-Fidelity PCR polymerase, New England Biolabs) and purified. Fragments were individually cloned into the pENTR/D-TOPO vector and subcloned in *E. coli*. Single colony plasmids were purified, and plasmid DNA restricted followed by Sanger sequencing (Macrogen). Confirmed inserts were introduced into pGWB405 destination vectors using the Gateway system. Primers and vectors are provided in Table S1. Final *35S:BvMLP* constructs to generate over-expressor (OE) lines were transformed into *Agrobacterium tumefaciens* strain C58, followed by transformation to *A. thaliana* Col-0 using the floral dip method (Davis et al. 2009). Twenty putative T_0_ transgenic plant lines for each construct were produced followed by in vitro selection for kanamycin resistance, and PCR analysis. Two independent, homozygous T_2_ lines per construct were chosen and propagated to generation T_3_ to amplify enough seeds for further analysis. Following *A. thaliana* materials were used in the study: *35S:BvMLP1-1* (OE1a), 3*5S*:*BvMLP1-2* (OE1b), *35S:BvMLP2-1* (OE2a), *35S:BvMLP2-2* (OE2b), *35S*:*BvMLP3-1* (OE3a) and *35S:BvMLP3-2* (OE3b). Homozygous single T-DNA insertion lines: *Atmlp1-1* (SALK 018534), *Atmlp2-1* (WiscdsLox413-416K24), *Atmlp3-1* (SALK_103714C), *Atmlp3-2* (SALK_033347C) and two double mutants *Atmlp1-1*/*Atmlp3-1* and *Atmlp1-1*/*Atmlp3-2* were also included in the work.

### Screening of sugar beet seedlings and Arabidopsis plantlets

*R. solani* AG2-2IIIB inoculum of BBA 69670 was prepared by growing fresh hyphae from a 1 cm^2^ potato dextrose agar plug for 10 days on sterile maize flour medium (1:1:5 ratio of maize flour, perlite and water). Three-week-old sugar beet seedlings of the four breeding lines were grown in standard soil followed by transfer to the growth containers with a mixture of fresh soil and prepared inoculum in a ratio of 10:1. At least five roots including hypocotyls were sampled in four biological replicates at 0, 2 and 5 dpi for each of the four sugar beet breeding lines. *A. thaliana* plantlets were transferred to containers containing a 20:1 ratio of fresh soil and inoculum after cultivation in standard soil for 21 days. Six biological replicates per genotype, each comprising of at least four plants were harvested at 5 dpi. All sugar beet and *A. thaliana* plants including wildtype Col-0 were grown under short-day conditions (8/16 h light/dark, 22/18 °C day/night).

### Fungal DNA quantification and *MLP* transcript analysis

Total plant RNA was extracted and cDNA synthesis was performed as earlier described. Gene-specific primers were designed using Primer3 (Rozen and Skaletsky 2000) and expression normalized to the *TUBB4* (sugar beet) or *Ubiquitin10* (*A. thaliana*) genes. Transcript data were analyzed with the comparative C_T_ method (Livak and Schmittgen 2001) followed by Student’s *t *test in R (version 3.16). Total DNA was extracted from inoculated samples (Möller et al. 1992). 500 µl of 3% CTAB extraction buffer per 100 mg disrupted plant material was used. The amount of fungal DNA (*RsG3PDH*) was determined with qPCR and normalized to the amount of plant DNA (*Actin2*). Primers are listed in Table S2.

### Availability of data and materials

RNA-Seq data have been deposited in the National Center for Biotechnology Information (NCBI) database, and Gene Expression Omnibus under the accession number GSE92859. The sugar beet genome RefBeet 1.0, used for the mapping is converted to the RefBeet 1.1, available at https://bvseq.molgen.mpg.de/index.shtml and translations can be seen in the processed data file.

## Results

### Three disease resistance-type genes are expressed as an early response to *R. solani*

**Fig. 1:**
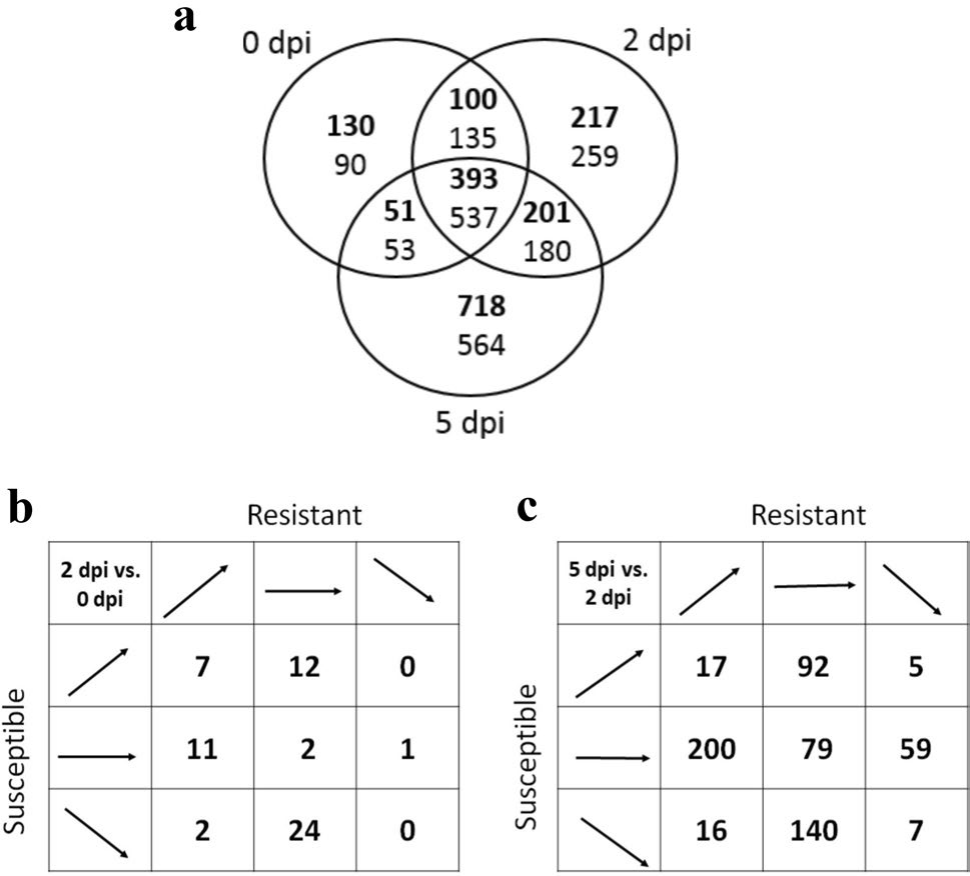
Differentially expressed sugar beet genes. **a** Partially resistant and susceptible genotypes were compared at three time points; 0, 2, and 5 days post-inoculation (dpi) with *R. solani*. Bold numbers represent up-regulated genes and numbers in regular text are down-regulated genes in partially resistant genotypes. The edgeR package (Robinson et al. 2010) was used for the analysis with absolute log2 fold change > 1 and false discovery rate < 0.05. **b** Significantly differentially expressed sugar beet genes comparing genotypes and time points. 2 dpi is compared with 0 dpi, a total of 59 genes, **c** 5 dpi is compared with 2 dpi, a total of 615 genes. Arrows indicate significant up- or down-regulation or no significant differential expression between time-points. The analysis was done using the R package edgeR (McCarthy et al. 2012) with absolute log2 fold change > 1 and false discovery rate < 0.05 settings

Sugar beet transcript data were generated for 47,713 gene models. A cut-off value was set at > five reads in at least three samples to avoid singleton bias, resulting in a final set of 16,768 genes for further analyzes. The major sources of variance in the data set were analyzed using principal component analysis. This variance was best explained by time post-inoculation followed by *R. solani* resistance level in sugar beet (Fig. S1). Data from the two partial resistant and the two susceptible genotypes were fused because no major source of variance was observed between them. This approach added statistical power to the tests of differential expression. Differential expression of the 16,768 genes was determined using a generalized linear model likelihood ratio test. During the time-course from day zero to 5 dpi, an overall increase of transcriptionally affected genes was found in partially resistant compared to susceptible genotypes (Fig. 1a). Gene ontology (GO) enrichment analysis did not find biological processes related to biotic stress over-represented in the list of genes up-regulated at 5 dpi (718) or those shared at 2 and 5 dpi (201). In contrast, 11 genes annotated as response to stress (GO term GO:0006950) were identified among the 217 transcripts up-regulated at the earlier time-point (2 dpi). Genes in this group were Bv1_007570_oxfa (abscisic stress-ripening (ASR) protein), Bv1_013700_wnij (peroxidase), Bv2_026070_scpc (unknown), Bv4_088600_cumk (NBS-LRR-type resistance protein), Bv7_178870_rzzu (peroxidase), Bv7_179080_rdtw (cationic peroxidase), Bv8u_204980_frqg (BED finger-NBS-LRR resistance protein), Bv9_206760_padn (rRNA N-glycosidase), Bv_25520_psek (peroxidase), Bv_44840_iifo (NBS-LRR-type resistance protein) and Bv2_039610_pxtp (unknown). The data suggest an early effect of three resistance-like genes to *R. solani* infection. They are Bv_44840_iifo, Bv4_088600_cumk and Bv8u_204980_frqg and located on chromosome 3, 4 and 8, respectively. These genes, encoding nucleotide-binding site and leucine-rich repeat (NBS-LRR) domains, were not significantly elevated at 5 dpi.

### Major latex protein-like protein encoding genes are activated in response to *R. solani* infection

To further clarify the influence of the infection-time component, a statistical test was performed to identify interaction effects between genotype and time after inoculation. In total, 660 genes were significantly different (false discovery rate (FDR) < 0.05) between partially resistant and susceptible genotypes in their response to *R. solani* inoculation (Fig. S2) Next, this set of genes was divided into functional groups using eukaryotic orthologous group (KOG) assignments. Out of the 660 genes, only 4 genes were assigned to defense mechanisms not seen in the GO enrichment analysis. Nine genes were annotated as cell wall-related genes (Table S3).

Early in the infection process (2 dpi vs. 0 dpi) 59 genes showed a significant differential response in partially resistant compared to susceptible genotypes (Fig. 1b), while the number increased at the later comparison (5 dpi vs. 2 dpi) to 615 (Fig. 1c). GO enrichment analysis showed that oxidation–reduction process (GO:0055114) genes were enriched at 2 dpi (Table S4). At 5 dpi, 19 GO groups were enriched including cell wall macromolecule catabolic process (GO:0016998), cellulose biosynthetic process (GO:0030244) and response to biotic stimulus (GO:0009607) (Table S5). In the latter GO group the three genes Bv7_162510_pymu, Bv7_162520_etow, and Bv_27270_xeas on chromosome 7 and 8, were annotated as major latex protein-like encoding genes (Table S6). Elevated levels of these three *MLP* genes, denoted as *BvMLP1*, *BvMLP2* and *BvMLP3*, were found in the partially resistant genotypes after 5 days of fungal challenge (Fig. S4). We further constructed a weighted gene co-expression network (Langfelder and Horvath 2008, 2012). A clustering of the weighted correlation network resulted in 48 modules with highly co-expressed genes (Table S7). GO enrichment analysis was performed on the genes with high correlation to each module (Data set S1). Modules 3, 4, 5, 18, 23 and 30 contained an over-representation of genes annotated as biotic stress-related genes, whereas cell wall-related genes were enriched in modules 1, 41 and 47. Out of these two main categories, only module 5 contained significantly differentially expressed biotic stress-related genes in the partially resistant genotypes in response to *R. solani*. Again, the same three *BvMLP* genes as in the GO enrichment analysis were identified.

**Fig. 2:**
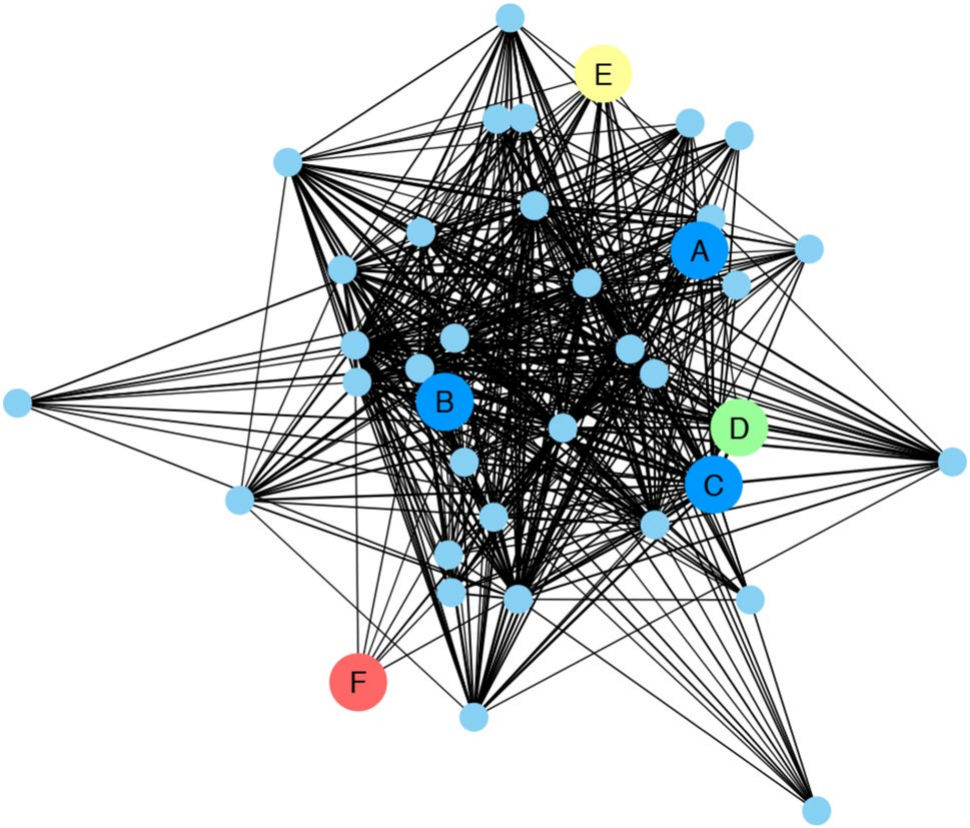
Co-expression network of differentially expressed sugar beet genes in module 5. The network comprises 38 genes (nodes) where blue represents: major latex protein homologs (A, BvMLP2; B, BvMLP1; C, BvMLP3), green (D): MYB46, yellow (E): flavonoid, red (F): disease resistance response protein and light blue represents other or unknown genes. Edge weight cut-off was set at > 0.16

In addition to *MLPs*, differentially expressed genes in module 5 included a MYB46 transcription factor (TF), a plant disease resistance response protein (DRR206) and a flavonoid *O*-methyltransferase protein, which are known to be involved in various stress response processes (Fig. 2). Two additional putative transcription factors, Bv2_027430_cint and Bv5_119300_wnjc, were significantly activated in the partially resistant genotype at 2 dpi, in contrast to the susceptible genotypes. These putative TFs were members of modules 6 and 14 of the co-expression network. In module 14, Bv2_027430_cint, an asymmetric leaf 2 (AS2) homolog, known as a repressive regulator, is highly correlated with six cell wall-related genes and five biotic stress-related genes significantly expressed at 5 dpi (Supplementary Table S8). In module 6, Bv5_119300_wnjc, a member of the APETALA2/Ethylene Responsive Factor (AP2/ERF) superfamily which regulates diverse plant responses, is connected with two biotic stress-related genes and two cell wall-related genes.

### *MLP1* and *MLP3* contributes to *R. solani* plant defense

**Fig. 3:**
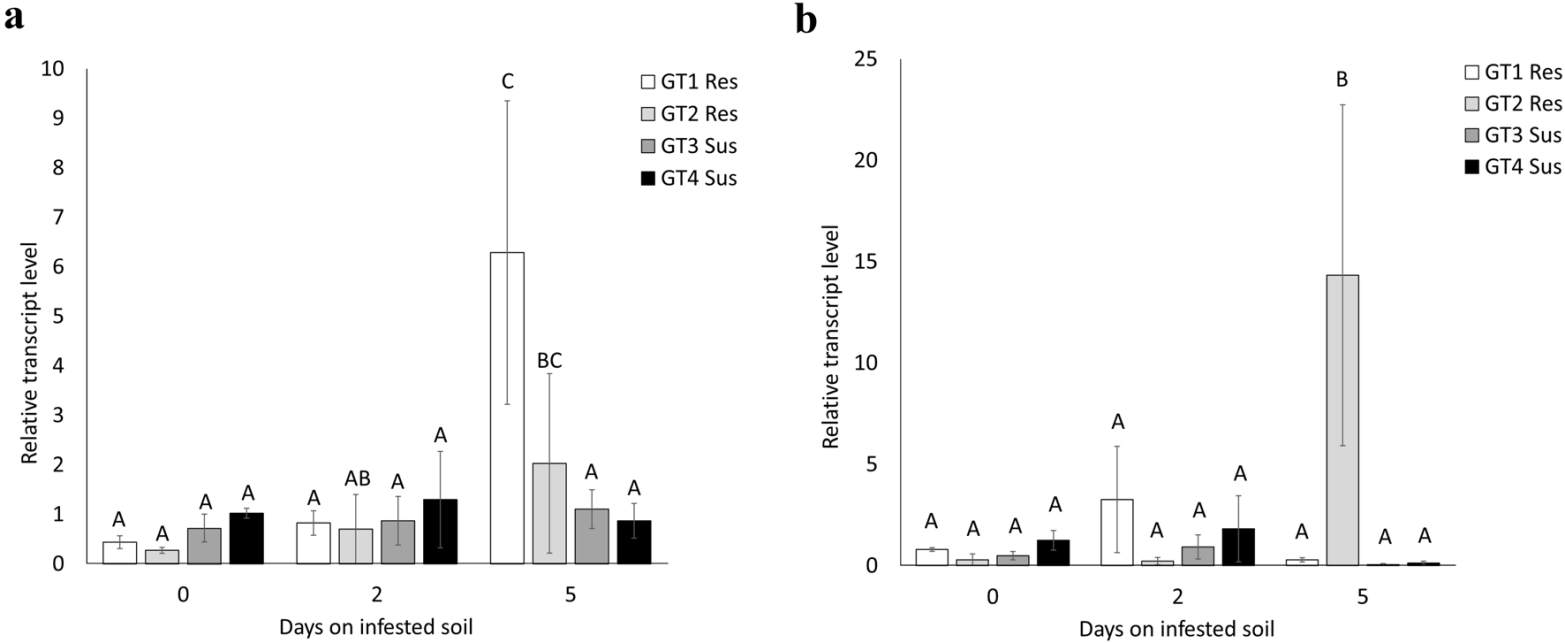
Relative transcript levels of *BvMLP* genes in sugar beet. Seedlings of four genotypes were harvested for real-time qRT-PCR at 0, 2 and 5 days on infested soil.** a** *BvMLP1,* **b** *BvMLP3*. The statistics are based on a Levene’s test and a Student’s *t *test on three biological replicates. Different letters indicate significant difference between groups. Error bars = mean ± SD

**Fig. 4:**
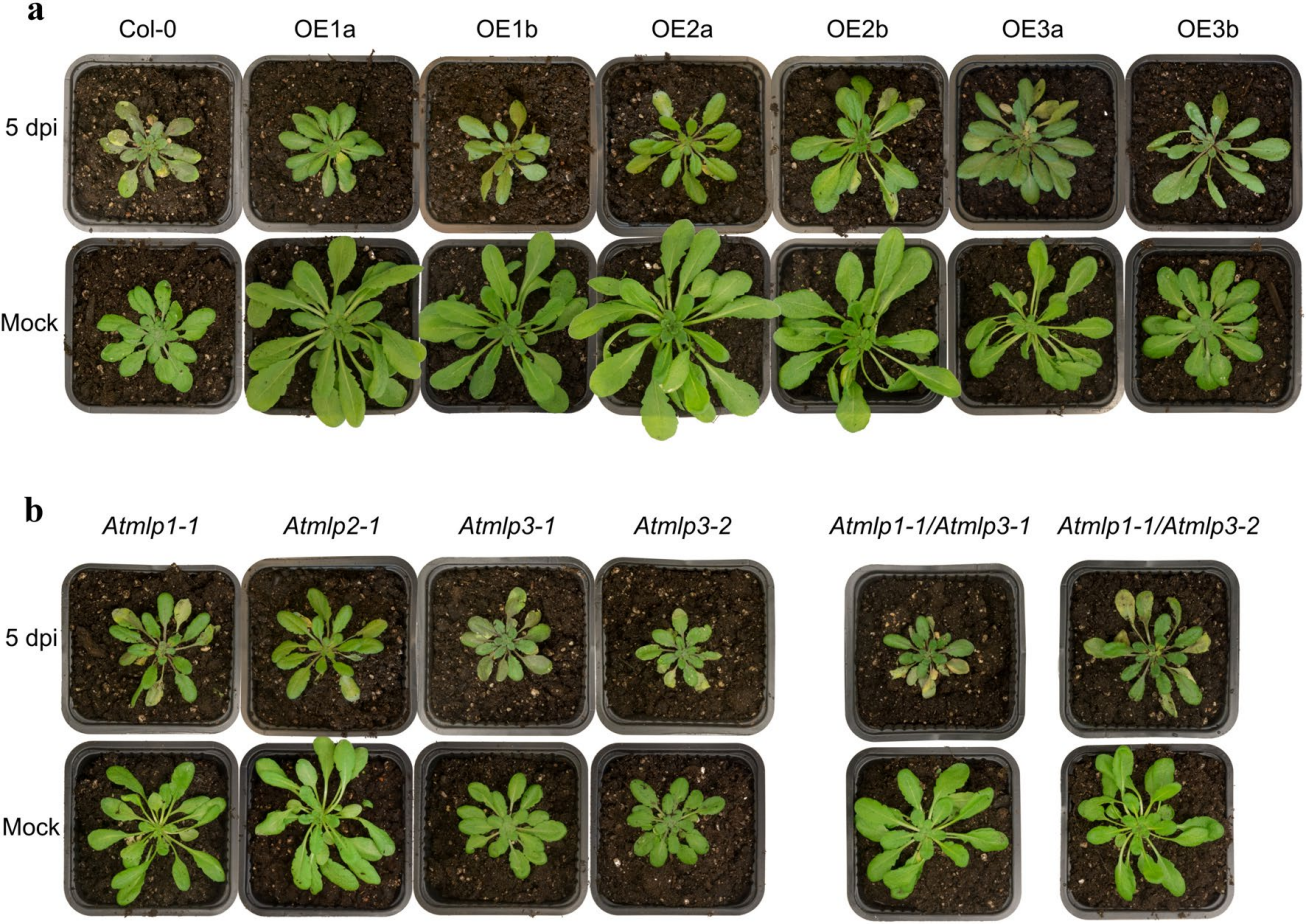
Phenotypes of *A. thaliana* inoculated with *R. solani* or H_2_O (mock). **a** Overexpressor lines: OE1a,b = *35S:BvML1-1*, *35S:BvML1-2*, OE2a,b = *BvML2-1*, 35S*:BvML2-2*, OE3a,b = 35S:*BvML3-1*, 35S*:BvML3-2.* **b** T-DNA insertion mutants in *BvMLP* homologues genes. Single mutants: *Atmlp1-1*, *Atmlp2-1*, *Atmlp3-1, Atmlp3-2,* and double mutants: *Atmlp1-1/Atmlp3-1* and *Atmlp1-1/Atmlp3-2*. All materials in Col-0 background. Photos taken 5 days post-inoculation

**Fig. 5:**
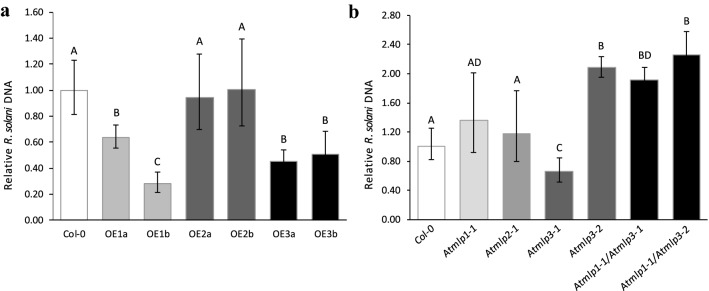
Relative amount of *R. solani* DNA in *A. thaliana*. **a** Two independent *BvMLP* overexpression lines per gene, and **b** *Atmlp* single mutant and double mutant lines compared to wild-type (Col-0) at 5 days post-inoculation. OE1a and OE1b = *35S:BvMLP1-1*, *35S:BvMLP1-2*, OE2a and OE2b = *BvMLP2-1*, *35S:BvMLP2-2*, OE3a and OE3b = *35S:BvMLP3-1*, *35S:BvMLP3-2*. Statistical analysis performed with a Student’s *t* test with at least four replicates. Error bars = mean ± SE

To confirm the prediction based on the RNAseq data, qRT-PCR analysis was performed on infested sugar beet seedlings. Significant differences in transcript responses were found at 5 dpi in young sugar beet seedlings for *BvMLP1* in genotype G1 and *BvMLP3* in genotype G2, both harboring partial resistance to *R. solani* (Fig. 3). No significant response was found for *BvMLP2* (Fig. S5). To further dissect the different contributions of the *BvMLP* genes, the three coding sequences were cloned from genotype G1 and overexpressed in *A. thaliana* (Fig. S6a). In parallel, homozygous T-DNA insertion mutants in homologous *A. thaliana* genes (At5g28010, At1g23130 and At1g70890) were produced (Fig. S6b, c). These *A. thaliana* genes shared 47%, 33% and 61% amino acid sequence identity to the three sugar beet genes *BvMLP1*, *BvMLP2* and *BvMLP3*, respectively. All MLP overexpression lines developed faster and formed larger rosettes than wild type (Col-0). After 5dpi, the *A. thaliana* transgenic and mutant lines were evaluated for responses to *R. solani* (Fig. 4a, b). When comparing the fungal DNA content in the different *A. thaliana* genotypes, *35S:BvMLP1-1*, *35S:BvMLP1-2* and *35S:BvMLP3-1* and *35S:BvMLP3-2* had significantly lower levels compared to Col-0, *35S:BvMLP2-1*, and *35S:BvMLP2-2* (Fig. 5a). When analyzing the T-DNA mutants, *Atmlp3-2* (*BvMLP3* homolog) showed the highest levels of *R. solani* DNA compared to Col-0 followed by *Atmlp1-1* (*BvMLP1* homolog). To clarify potential redundancy effects of the two *AtMLP* homologs*,* two double mutants (*Atmlp1-1*/*Atmlp3-1* and *Atmlp1-1/Atmlp3-2*) were made and screened against *R. solani* (Fig. 4b). Fungal DNA analysis demonstrated higher levels in *Atmlp1-1/Atmlp3-2* than in the *Atmlp1-1* and *Atmlp3-1* single mutants (Fig. 5b). Together the data suggest that the *Atmlp3-2* mutation has the largest impact but *Atmlp1-1* add some strength to the response.

## Discussion

Today’s sugar beet cultivars with high levels of resistance to *R. solani* are known to suffer from yield penalty or harbor less resistance to other important pathogens (Strausbaugh et al. 2013; Liu and Khan 2016). We, therefore, were interested to monitor transcript responses to this fungus on a genome-wide scale to identify defense-associated genes useful to refine the breeding work. Our transcriptome profiling identified in total 2022 differentially expressed genes at 2 dpi and slightly more (2697) at 5 dpi in the dataset. GO enrichment analysis revealed eleven defense-associated genes differentially expressed at 2 dpi. Three genes containing NBS-LRR domains characteristic for resistance *R* genes were found among the genes expressed early, all three located on chromosome 3. QTL mapping has earlier identified two major clusters of NBS-BACs on chromosome 3 (Lein et al. 2008). This quantitative *R. solani* resistance coverers 10–15% of the sugar beet genome and is associated with negative yield drag. In attempts to further optimize the breeding work, additional gene candidates were searched for.

By exploiting interaction statistics, three *MLP* like-encoding genes were identified in the partially resistant genotypes, all being increasingly activated by time. Present in all MLP proteins is a ligand-binding site for large hydrophobic molecules, hormones and secondary metabolites that allow MLPs to have multiple functions (Koistinen et al. 2005; Ma et al. 2009; Park et al. 2009). MLPs are associated with tolerance to salt and drought in *A. thaliana* (Chen and Dai 2010; Wang et al. 2016) and are activated in response to the *Alternaria brassicicola* fungus and the soil-borne plasmodiophorid *Plasmodiophora brassicae* (Schenk et al. 2000; Siemens et al. 2006). *Verticillium dahliae* is another soil-borne pathogen (ascomycete) with a broad host range that includes cotton, sugar beet and many other species (Peggy and Brady 2002). In case of cotton, the *GhMLP28* was found to enhance the activity of an ethylene response factor, *GhERF6* and thereby amplified the defense response (Yang et al. 2015). No co-activation of *ERF*-encoding genes in our sugar beet datasets was observed. The number of MLP-like proteins varies among plant species (Zhang et al. 2018). A trend seen so far is that fewer homologs are found in monocots compared to dicots. 23 *MLP* genes are present in the sugar beet genome compared to 25 in *A. thaliana*. In attempts to dissect the importance of the three *BvMLP* genes in the response to *R. solani*, we first analyzed each *BvMLP* gene independently. RNAseq gene expression levels were confirmed with qRT-PCR for two of the *MLP* genes (*BvMLP1* and *BvMLP3*). *BvMLP1 and BvMLP3* individually contributed to reduced infection levels of *R. solani* when overexpressed in *A. thaliana.* When pathogen responses of T-DNA insertion mutants in the most homologous *MLP* genes in *A. thaliana* were monitored the *Atmlp3-2* mutant and the *Atmlp1-1/Atmlp3-2* double mutant yielded the highest level of infection. The data suggest that both *BvMLP1* and *BvMLP3* should be integrated in resistance breeding approaches to *R. solani*.

The genome of *R. solani* is enriched in genes coding for carbohydrate cell wall-degrading enzymes (Wibberg et al. 2016). This knowledge formed the rational to also include genes important for cell wall biogenesis in the analysis. Several TFs are known to regulate secondary cell wall formation. Among those, *MYB46* has a key function involving biosynthesis of cellulose, hemicellulose and lignin components (Ko et al. 2014). *MYB46* was clearly activated in the present sugar beet transcripts. In the sugar beet genome, as in *A. thaliana*, only one *MYB46* gene together with its paralogue *MYB83* is present. *MYB46* homologues in poplar, maize and rice are known to possess similar function in secondary wall biosynthesis as in *A. thaliana* (Zhong et al. 2010, 2011), which leads us to believe that this function is conserved also in sugar beet. The *DRR206* gene is well studied in pea, where it is activated both in response to bacterial and fungal infections (Daniels et al. 1987). *DRR206* expression is associated with pathways involving phytoalexins and cell wall biosynthesis (Hadwiger and Chang 2015; Seneviratne et al. 2015). Interestingly transgenic *Brassica napus* plants harboring the overexpressed pea *DRR206* gene showed enhanced seedling resistance to *R. solani* (Wang and Fristensky 2001). Together these data suggest that an activated *DRR206* gene may contribute to defense in sugar beet.

Plant carbohydrate metabolism is involved in numerous processes including cell wall structure, cell shape, energy metabolism, post-translational modifications, signaling, and defense (Kubicek et al. 2014). The cell wall composition and architecture affect wall strength, which forms an important physical outer barrier to potential invading pathogens. A common theme of fungal plant pathogens is their ability to secrete cell wall-degrading enzymes (Kubicek et al. 2014). The *R. solani* AG2-2IIIB isolate BBA 69670 that preferentially attacks sugar beets is no exception and encodes a wide repertoire of carbohydrate active enzymes (Wibberg et al. 2016). Particularly, glycoside hydrolase 43 (GH-43), carbohydrate esterase 12 (CE-12) and polysaccharide lyases 1 (PL-1) families are enriched in this fungal genome. In the sugar beet genome, we found 1294 CAZyme-encoding genes and 1349 CAZyme annotated domains which are slightly higher compared to the 1200 CAZy annotated proteins in *A. thaliana* (Fig. S7). Small proportions of the CAZyme domain classes were differentially expressed during fungal challenge. In comparison to *A. thaliana*, sugar beet has fewer glycosyl transferases (GT) and about the same numbers of glycoside hydrolases (GH), carbohydrate-binding modules (CBM) and polysaccharide lyases (PL). However, an enrichment of carbohydrate esterases (CE) and particularly large numbers of auxiliary activities (AA) are annotated in the sugar beet genome compared to *A. thaliana.* Most of these AA proteins belong to the AA2 family. This family contains class II lignin-modifying peroxidases that oxidize Mn(II) to Mn(III) which in turn oxidize a variety of phenolic model compounds able to degrade and or modify lignin polymers (Levasseur et al. 2013).

In conclusion, monitoring plant responses to soil-borne pathogens is challenging due to their hidden life in the soil which is difficult to control and observe. To this end, knowledge on their modes of infection and external factors impacting the infection process is low. *Rhizoctonia solani* is no exception where disease symptoms, if seen, are represented by dead plants on heavily infested soil. Our present study has highlighted a number of gene families that could contribute to *R. solani* defense in sugar beet, maybe in an orchestrated fashion during the fungal attack and disease progression. Any biotrophic stage of *R. solani* has so far not been demonstrated but early involvement of *R*-genes may be a sign of a hemibiotrophic lifestyle. Likewise, *R. solani* produces a chitin-binding LysM effector perturbing chitin-induced immunity which adds further support to a possible presence of an initial biotrophic infection stage (Dölfors et al. 2019). *Rhizoctonia solani* has a large repertoire of carbohydrate-active enzyme (CAZy)-encoding genes in its genome suitable for cell wall degradation, important for necrotrophic growth and saprophytic survival. Involvement of *MLP* genes are observed as a plant response to other soil-borne fungi such as *V. dahliae* (Yang et al. 2015). Its function to fungal invasion is still unclear. Recently, in an RNAseq study of fungus–apple interaction, one *MLP* gene was found to impact a handful of defense-related genes including transcription factors (He et al. 2020). It seems that *MLP* genes play important roles for defense in many crops including sugar beet; details of their function remain to be elucidated.

## Electronic supplementary material

Below is the link to the electronic supplementary material.

Supplementary file1 (DOCX 1776 kb)
