# Supplementary material for: Major latex protein-like encoding genes contribute to Rhizoctonia solani defense responses in sugar beet
Source: Mol Genet Genomics. 2020 Oct 28;296(1):155–64. doi: 10.1007/s00438-020-01735-0 (PMC7840631; doi:10.1007/s00438-020-01735-0)

**Supplementary material**

**Major latex protein-like encoding genes contribute to *Rhizoctonia solani* defense responses in sugar beet**

Louise Holmquist^1,2§^, Fredrik Dölfors^2§^, Johan Fogelqvist^2^, Jonathan Cohn^3^, Thomas Kraft^1^ and Christina Dixelius^2*^

^1^MariboHilleshög Research AB, Säbyholmsvägen 24, S-26191 Landskrona, Sweden

^2^Swedish University of Agricultural Sciences, Department of Plant Biology, Uppsala BioCenter, Linnean Center for Plant Biology, P.O. Box 7080, S-75007 Uppsala, Sweden

^3^Syngenta, Crop Protection. 9 Davis Drive, Research Triangle Park, NC 27709, USA

**Content**

Fig. S1 to Fig S7. Table S1 to Table S8

**
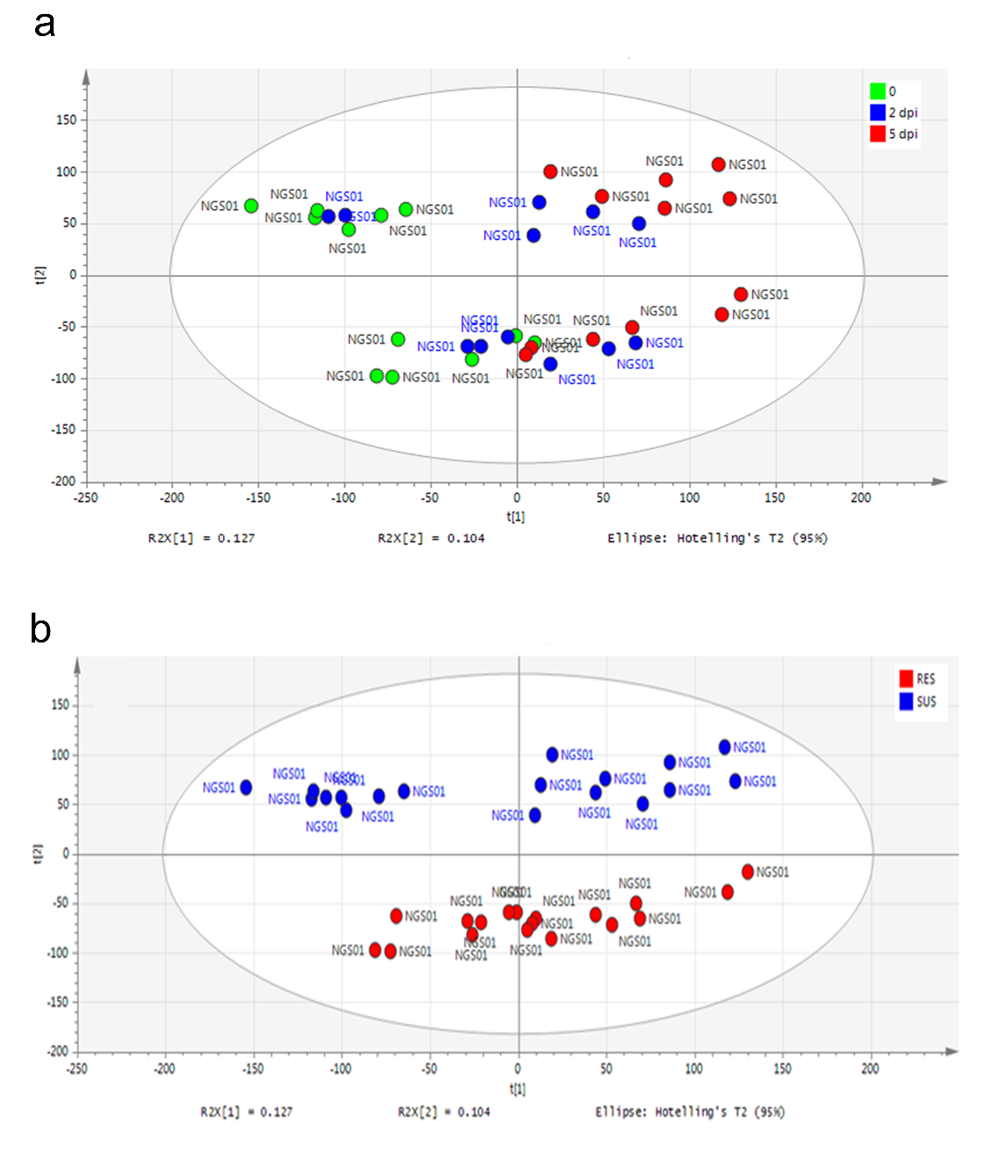
**

**Fig. S1** Principal component analysis (PCA) plots created in Simca version 13.0.0 (http://umetrics.com/products/simca). **a.** PC1 time post inoculation. **b.** PC2 Genetic background.


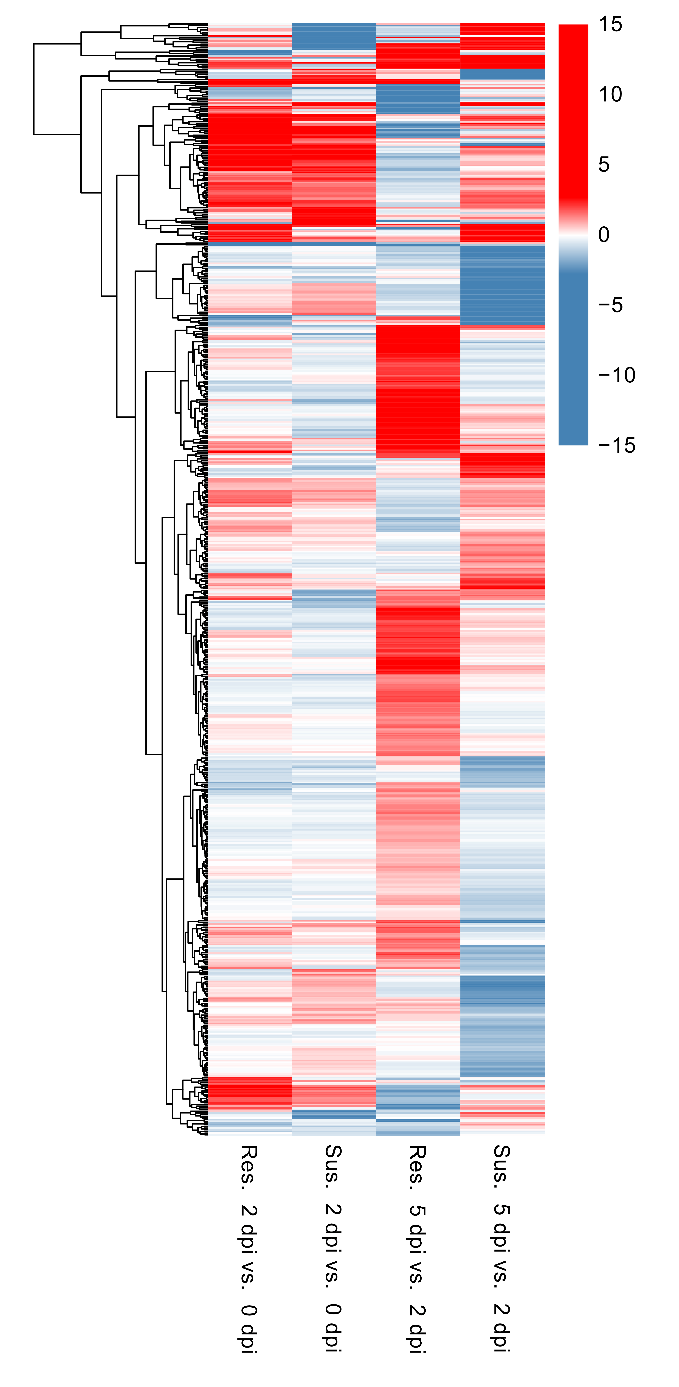


**Fig. S2** Heat map visualizing 660 differentially expressed genes. Two time-points; early (2 dpi vs. 0 dpi) and late (5 dpi vs. 2 dpi) were compared for the partially resistant and susceptible sugar beet genotypes. Absolute log2 fold change > 1 and false discovery rate (FDR) < 0.05. Log2 fold change values are truncated at 5 and -5 for a clearer visualization.

**
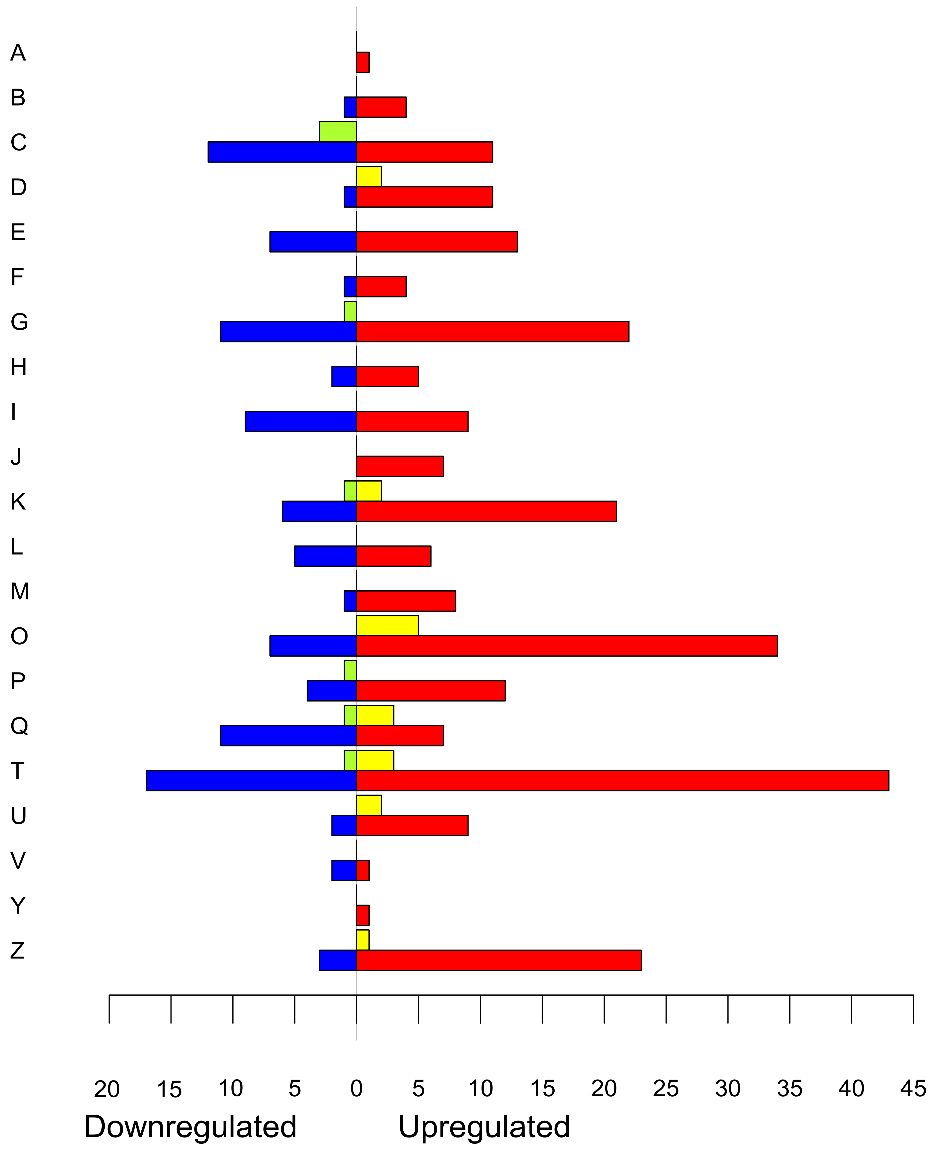
**

**Fig. S3** Differentially expressed sugar beet genes distributed among functional groups (KOG). 369 genes with unknown KOG function are excluded. The plot is based on the remaining 291 genes. Color codes: yellow; up-regulated genes at 2 dpi vs. 0 dpi, red; up-regulated genes at 5 dpi vs. 2 dpi, green; down-regulated genes at 2 dpi vs. 0 dpi, and blue; down-regulated genes at 5 dpi vs. 2 dpi. A; RNA processing and modification B; Chromatin structure and dynamics C; Energy production and conversion D; Cell cycle control, cell division, chromosome partitioning E; Amino acid transport and metabolism F; Nucleotide transport and metabolism G; Carbohydrate transport and metabolism H; Coenzyme transport and metabolism I; Lipid transport and metabolism J; Translation, ribosomal structure and biogenesis K; Transcription L; Replication, recombination and repair M; Cell wall/membrane/envelope biogenesis O; Posttranslational modification, protein turnover, chaperones P; Inorganic ion transport and metabolism Q; Secondary metabolites biosynthesis, transport and catabolism T; Signal transduction mechanisms U; Intracellular trafficking, secretion, and vesicular transport V; Defense mechanisms Y; Nuclear structure Z; Cytoskeleton.


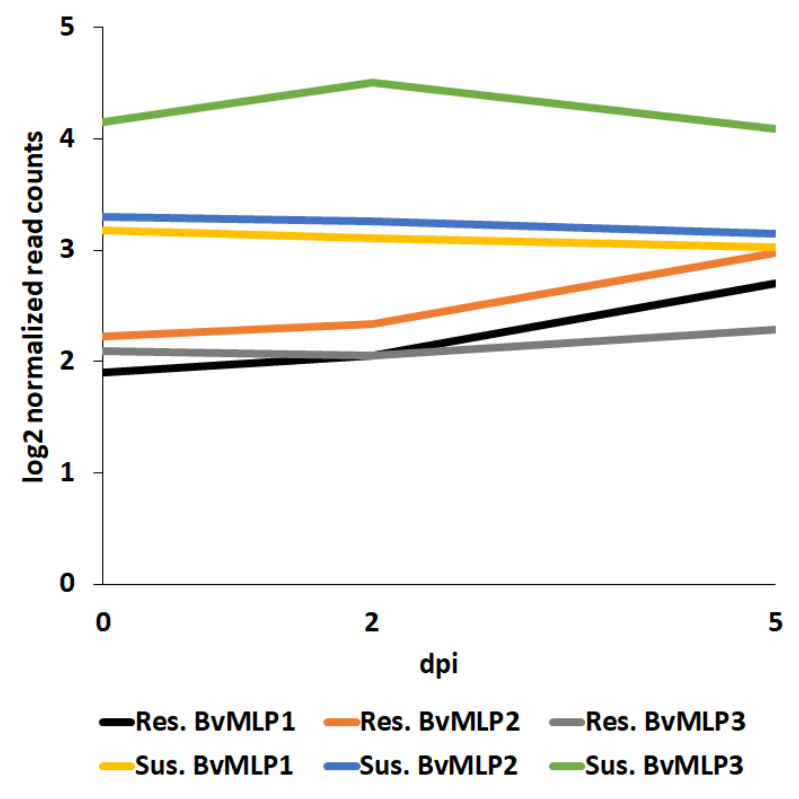


**Fig. S4** Expression pattern of three major latex protein gene homologs over time. The three genes *BvMLP1* (Bv7_162510_pymu), *BvMLP2* (Bv7_162520_etow) and *BvMLP3* (Bv_27270_xeas) were significantly differentially expressed (FDR < 0.05) in partially resistant (Res) compared to susceptible (Sus) genotypes at 5 days post inoculation (dpi) compared with 2 dpi. Data used for this graph was apportioned read counts, normalized for GC content and an average of six replicates.

**
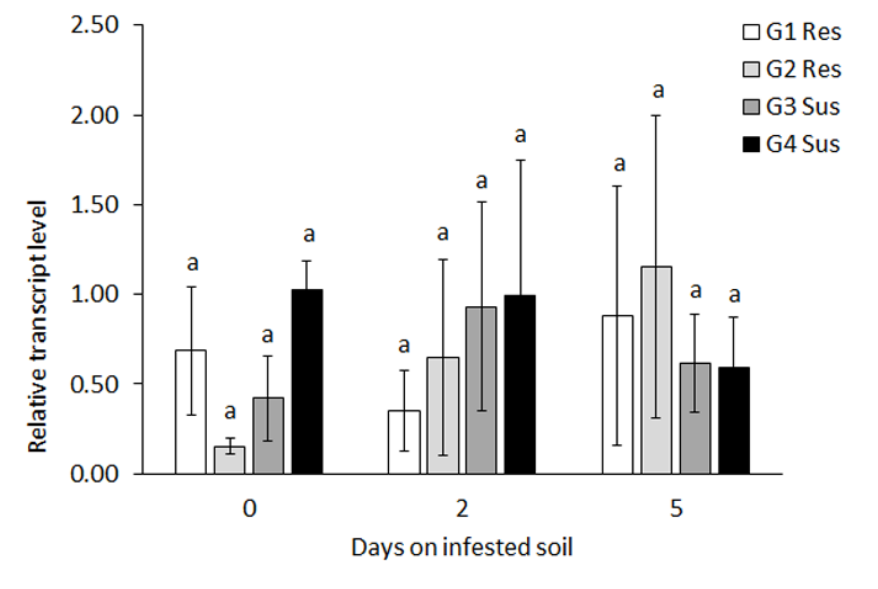
**

**Fig. S5** Relative transcript levels of *BvMLP2* gene in sugar beet. Seedlings of four genotypes were harvested for real-time qRT-PCR at 0, 2 and 5 days on infested soil. Statistics are based of three biological replicates followed by Levene's test and a Student t-test. Error bars = mean ± SD.


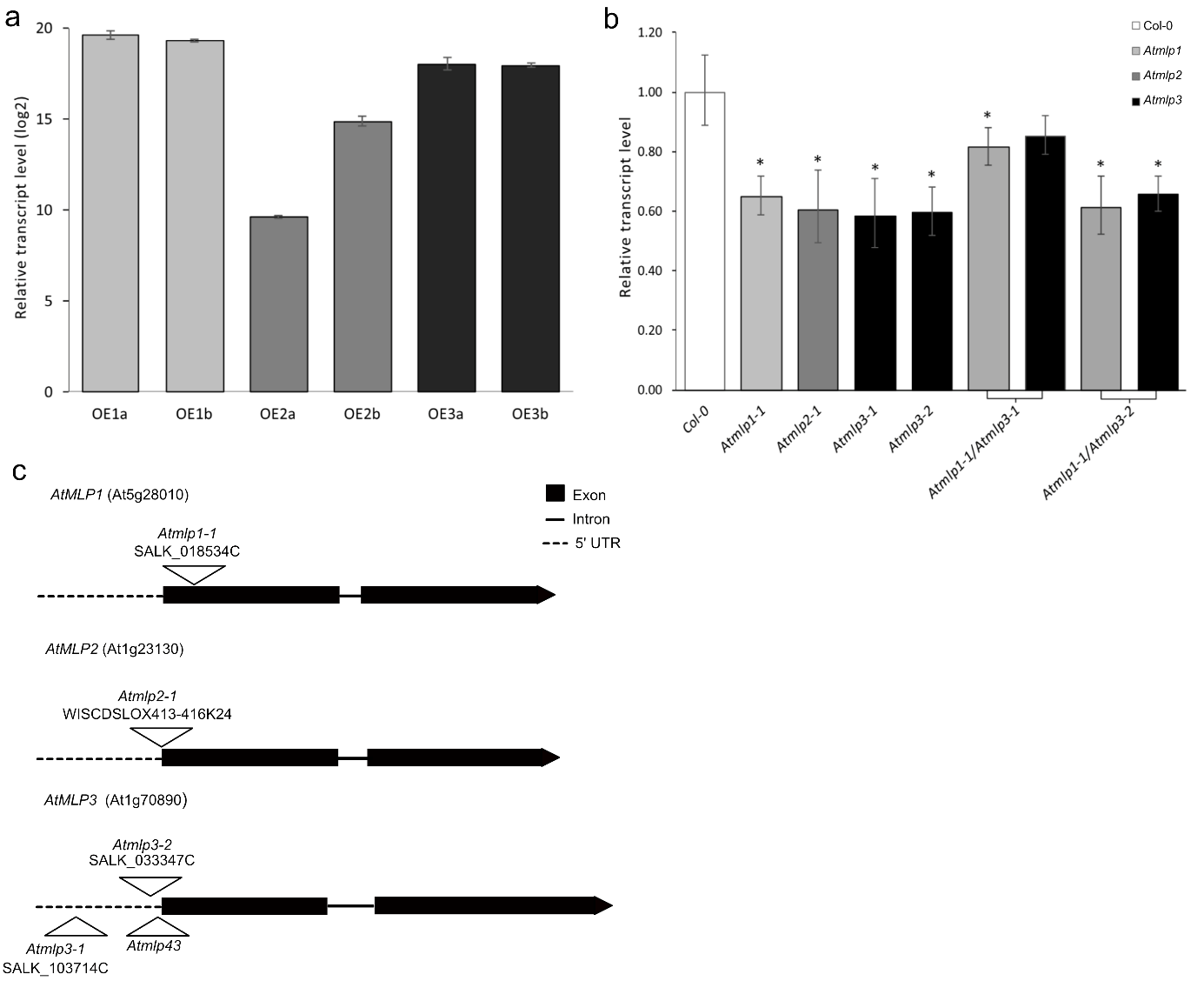


**Fig. S6** *A. thaliana* materials monitored for responses to *R. solani*. **a.** Relative transcript level of transgenic *35S:BvMLP*. **b.** Relative transcript level of *Atmlp* single and double mutant lines. Statistical analysis of transcript levels was performed using Student t-test on at least 3 biological replicates. Each genotype is normalized to *UBIQUITIN10* and relative to expression in Col-0. * = significant p-value with confidence level of at least α = 0.05, error bars = mean ± SE. **c.** Illustration of the T-DNA positions in *AtMLP1* (At5g28010), *AtMLP2* (At1g23130) and *AtMLP3* (At1g70890) (not in scale). *Atmlp43* denotes the T-DNA position of *Atmlp43* (At1g70890, SALK_109337) published by Wang et al. (2016). OE1a and OE1b = *35S:BvML1-1*, *35S:BvML1-2,* OE2a and OE2b = *BvML2-1*, *35S:BvML2-2*, OE3a and OE3b = *35S:BvML3-1*, *35S:BvML3-2*.

**
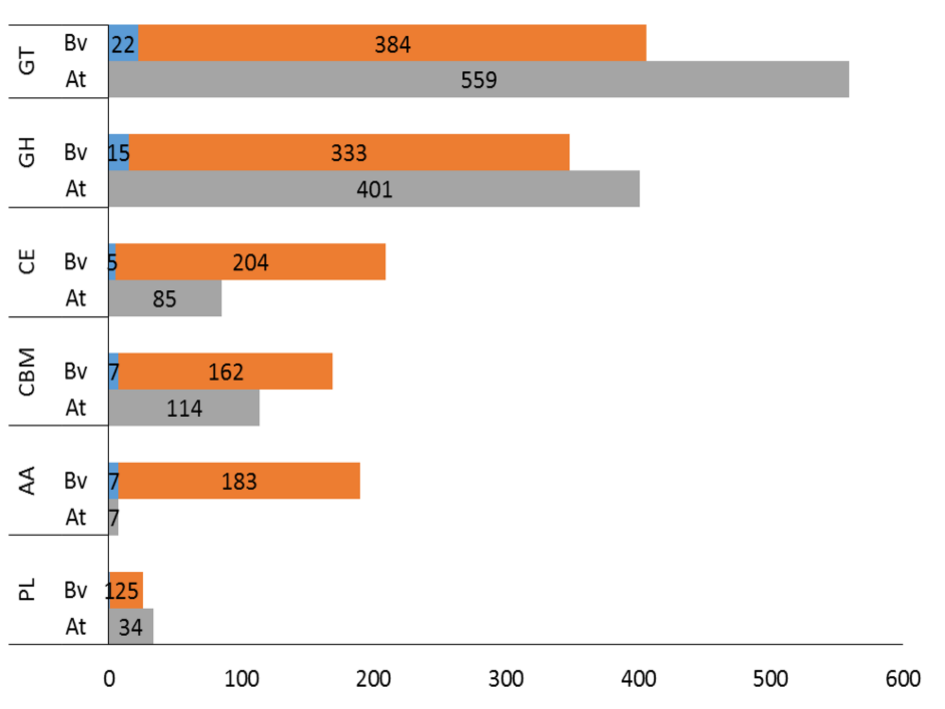
**

**Fig. S7** Distribution of CAZy domain annotated proteins in sugar beet (*B. vulgaris*, Bv) (orange) and *A. thaliana*, At, (grey) genomes distributed among CAZy class. Glycoside hydrolase (GH), Glycosyl transferase (GT), Polysaccharide Lyase (PL), Carbohydrate esterase (CE), Auxiliary activities (AA). Blue box represent number of differentially expressed sugar beet genes in each CAZy class.

**Table S1** Constructs, primers and vectors used for Agrobacterium-transformation of *A. thaliana* (Col-0).

| Construct | Primer sequence (5’ – 3’) | Entry vector | Destination vector |
| --- | --- | --- | --- |
| 35S:BvMLP1:GFP | F: CACCATGGCAGGTCTAAAGCGTAAGCT  R: AATTAACGAGTTTGAGCATGAATCA | pENTR/D-TOPO | pGWB405 |
| 35S:BvMLP2:GFP | F: CACCATGGCAGGTCTAAAGCGTAAGCT  R: TTTATCTTGGCAATGGTGATGA | pENTR/D-TOPO | pGWB405 |
| 35S:BvMLP3:GFP | F:CACCATGGGAGTGACAGGGAAGCTAGA  R: TGCTTTAAAATGATGATCCTCGA | pENTR/D-TOPO | pGWB405 |

**Table S2** Primers used for qPCR of *R. solani* biomass in *A. thaliana* *Atmlp* knockouts and *BvMLP* overexpressor lines, qRT-PCR primers to confirm knockout/overexpressor status in *A. thaliana* (Col-0) and qRT-PCR primers for *BvMLP* gene expression in *B. vulgaris.*

| Gene^a^/ Locus ID | Primer sequence (5’ – 3’) |
| --- | --- |
| *qPCR Primers:* |  |
| AtACT2/AT3G18780 | F: CTTGCACCAAGCAGCATGAA |
|  | R: CCGATCCAGACACTGTACTTCCTT |
| RsG3PDH/RSOLAG22IIIB_07022 | F: ACCGTTATGGGCTTGTCTTTCCTT |
|  | R: CCCGCTTGGCTCGAATAGTAACG |
| *qRT-PCR primers:* AtMLP1-1/At5g28010 | F: CAAGCAAAGGTTGCAAAAGA |
|  | R: AGAAAATTCTCGGGGTCAGG |
| AtMLP2-1/At1g23130 | F: ATGGCACAAGCTACGCGTCA |
|  | R: CTAGACTTCGGACAAAAGC |
| AtMLP3-1/At1g70890 | F: GGCAGTGGATCAGGAGAAGA |
|  | R: CGTCCATTGATTCGGTTAGC |
| AtMLP3-2/At1g70890 | F: GGCAGAAGCGTCTAGTTTGG |
|  | R: GACGATAGAGCCGACTTTGC |
| BvMLP1/Bv7_162510_pymu | F: AGAGATAAGAGTTGCAGGGGG  R: GCACCATCATGCAAGTCACAG |
| BvMLP2/Bv7_162520_etow | F: AGATGCTGGAAGGAGATTTGATGG  R: TCATCAATCTCTTTCGCCAGTCC |
| BvMLP3/Bv_27270_xeas | F: GGGAGTGACAGGGAAGCTAGA |
|  | R: TCACCCTCATGTGCGTTACA |
| UBIQ10/At4G05320  BvB-TUBULIN/ BVRB_4g074050 | F: AGGTACAGCGAGAGAAAGTAGCA  R: TAGGCATAGCGGCGAGGCGT  F: TGCTACCCTCTCCGTCCATCAGCTT R: ACCAGGGAACATCAGGCAGCAGG |

*^a^*At *= Arabidopsis thaliana* (Col-0)*,* Rs = *Rhizoctonia solani* AG2-2IIIB, Bv = *Beta vulgaris*

**Table S3** Defense and cell wall genes responding differently in partially resistant and susceptible genotypes to *R. solani.*


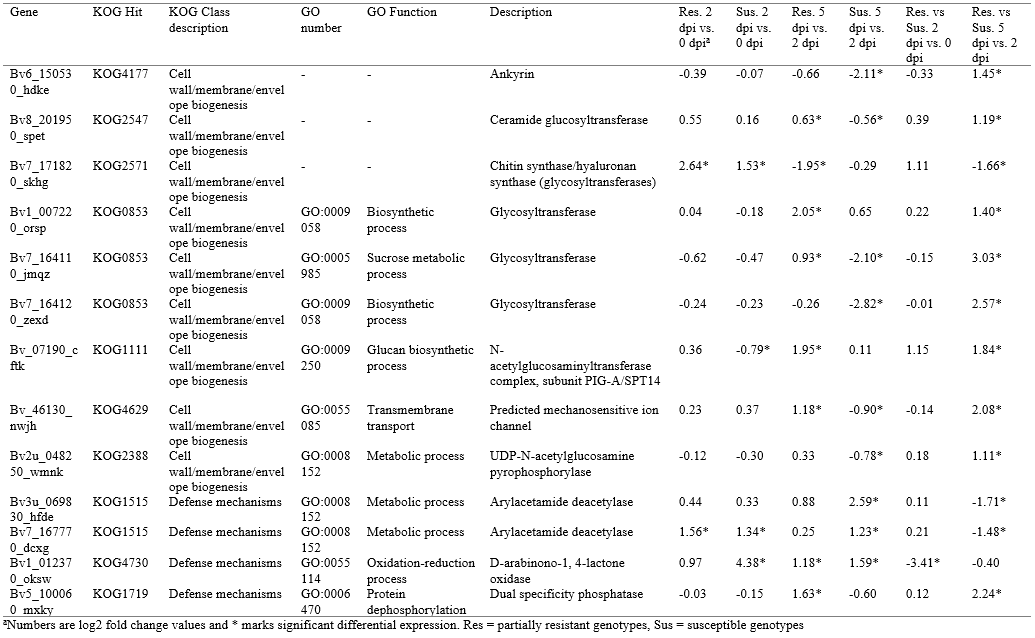


**Table S4** GO enrichment analysis of genes with different expression at 2 dpi compared with 0 dpi.

**Table S5** GO enrichment analysis of genes with different expression at 5 dpi compared with 2 dpi.

**Table S6** Cell wall and biotic stimulus related genes identified in GO enrichment analysis.


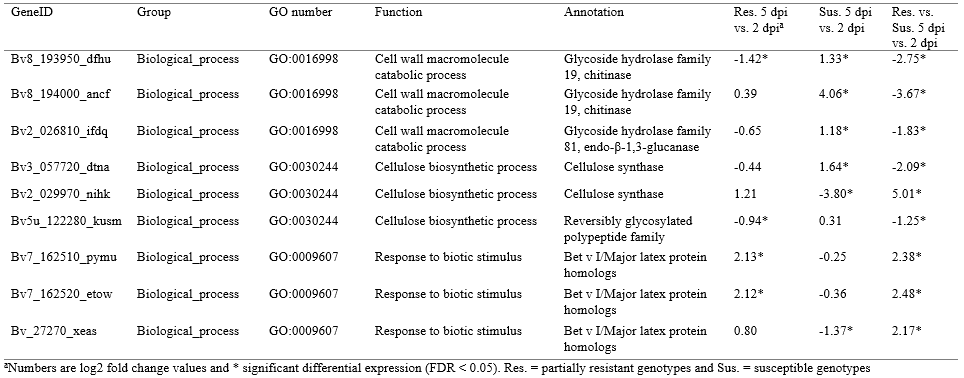


**Table S7** Number of genes distributed among modules for weighted correlation network.

| Module number | Module color | Number of genes in module |
| --- | --- | --- |
| 0 | grey | 664 |
| 1 | turquoise | 2017 |
| 2 | blue | 919 |
| 3 | brown | 913 |
| 4 | yellow | 698 |
| 5 | green | 693 |
| 6 | red | 686 |
| 7 | black | 674 |
| 8 | pink | 652 |
| 9 | magenta | 641 |
| 10 | purple | 531 |
| 11 | greenyellow | 514 |
| 12 | tan | 439 |
| 13 | salmon | 360 |
| 14 | cyan | 357 |
| 15 | midnightblue | 338 |
| 16 | lightcyan | 335 |
| 17 | grey60 | 307 |
| 18 | lightgreen | 245 |
| 19 | lightyellow | 244 |
| 20 | royalblue | 243 |
| 21 | darkred | 237 |
| 22 | darkgreen | 235 |
| 23 | darkgrey | 233 |
| 24 | darkturquoise | 233 |
| 25 | orange | 225 |
| 26 | darkorange | 222 |
| 27 | white | 222 |
| 28 | skyblue | 204 |
| 29 | saddlebrown | 193 |
| 30 | paleturquoise | 184 |
| 31 | steelblue | 184 |
| 32 | violet | 181 |
| 33 | darkolivegreen | 180 |
| 34 | darkmagenta | 171 |
| 35 | sienna3 | 170 |
| 36 | yellowgreen | 169 |
| 37 | skyblue3 | 168 |
| 38 | plum1 | 163 |
| 39 | orangered4 | 162 |
| 40 | mediumpurple3 | 98 |
| 41 | lightsteelblue1 | 89 |
| 42 | lightcyan1 | 65 |
| 43 | ivory | 64 |
| 44 | floralwhite | 62 |
| 45 | darkorange2 | 58 |
| 46 | bisque4 | 43 |
| 47 | brown4 | 43 |
| 48 | darkslateblue | 40 |

**Table S8** Cell-wall and biotic stress related genes connected with differential expressed transcription factors (TFs).


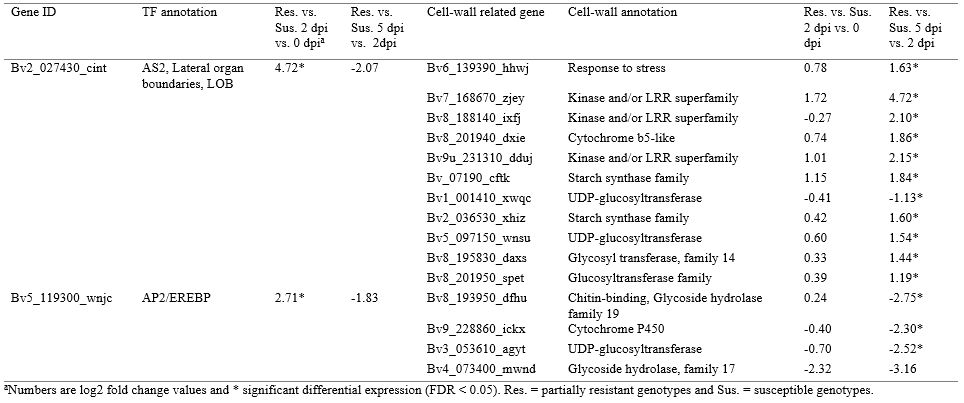

Supplement: Supplementary file 1 — Supplementary file1 (DOCX 1776 kb) [file 438_2020_1735_MOESM1_ESM.docx]
